# Acute Onset of Suicidal Ideation after Starting Lemborexant in a Patient with a Mood Disorder

**DOI:** 10.1055/s-0046-1829078

**Published:** 2026-10-09

**Authors:** Renato Rodrigo Silva, Marjorie Mastellaro Baruzzi, Bianca Besteti Fernandes Damiano, Rodolfo Furlan Damiano

**Affiliations:** 1 Program for Education, Research, and Care in Treatment-Resistant Depression, Self-Injury, and Suicidality, Hospital das Clínicas, Faculty of Medicine, University of São Paulo, São Paulo, Brazil; 2 Department and Institute of Psychiatry, Hospital das Clínicas, Faculty of Medicine, University of São Paulo, São Paulo, Brazil; 3 Private practice, Uberlândia, Minas Gerais, Brazil

**Keywords:** lemborexant, insomnia, suicidal ideation, dual orexin receptor antagonists, case report, sleep

## Abstract

A 24-year-old woman with treatment-resistant depressive symptoms and chronic insomnia developed acute, intrusive suicidal ideation with intent on day 5 after starting lemborexant 10 mg nightly. Emergency assessment did not identify intoxication, metabolic disturbance, psychosis, mania, akathisia, or other acute precipitants. Pretreatment polysomnography showed severe insomnia with a sleep efficiency of 14.5% and absent REM sleep, without sleep-disordered breathing. Lemborexant was discontinued immediately, and suicidal ideation resolved completely within 24 hours without additional pharmacological intervention. The Naranjo Adverse Drug Reaction Probability Scale yielded a score of 6, consistent with a probable adverse drug reaction. At 6-month follow-up, the patient remained free of suicidal ideation with sustained functional stability. This case adds clinically relevant documentation to pharmacovigilance concerns about potential suicidality with dual orexin receptor antagonists and supports structured suicide risk assessment and early follow-up when initiating these agents in patients with mood disorders.

## Introduction

Insomnia is common and clinically consequential, and longitudinal evidence links sleep disturbance, particularly insomnia, to later suicidal ideation and suicidal behaviors independent of depressive severity. 1 Dual orexin receptor antagonists (DORAs), including lemborexant, improve sleep by selectively blocking OX1R and OX2R receptors within the arousal network. 2 3 Randomized and long-term studies have suggested favorable efficacy and tolerability. 4 5 6 More recently, a 12-week open-label multicenter pilot study (SELENADE) reported improvements in sleep and depressive symptoms in patients with insomnia comorbid with depressive episodes, with no serious treatment-emergent adverse events; given its open-label pilot design ( *n* = 83), however, the study was not powered to detect rare psychiatric adverse events. 7 However, the United States prescribing information cautions about worsening depression. 2 We report a case of acute, severe suicidal ideation temporally associated with the initiation of lemborexant, followed by rapid and complete remission after drug discontinuation. Written informed consent for the publication of this case report, including anonymized clinical, polysomnographic, laboratory, and follow-up data, was obtained from the patient. The case is reported in accordance with the CARE guidelines for case reports. 8

## Case Presentation

A 24-year-old woman had a psychiatric history marked by treatment-resistant depressive symptoms and chronic insomnia since childhood. Several medication classes had provided only transient or partial benefit, including antidepressants, anticonvulsants, antipsychotics, and stimulants. She had also previously received ketamine, electroconvulsive therapy, and repetitive transcranial magnetic stimulation. By the time of the index exposure, the patient had failed adequate sequential trials of multiple classes of antidepressants and standard somatic and neuromodulation strategies for treatment-resistant depression, fulfilling commonly accepted criteria for treatment resistance. Insomnia, however, had remained the most refractory and clinically dominant symptom across the course of her illness, which was the rationale for initiating a DORA at this stage of treatment. Medical comorbidities included vasovagal dysautonomia and parasomnias, with historical restless legs symptoms and sleepwalking. Family history was notable for mood disorders and suicide.

Several months before the index exposure, evaluation for refractory insomnia showed unremarkable laboratory results. Pretreatment polysomnography recorded 71 minutes of total sleep over 491 minutes (sleep efficiency 14.5%), with sleep-onset latency of 297 minutes, stage distribution of N1 16.1%, N2 21.0%, and N3 62.9%, absent REM sleep, apnea-hypopnea index of 1.6/hour, and normal oximetry.

Because of persistent difficulty maintaining sleep, lemborexant 10 mg nightly was started. Concomitant medications, with their respective indications in this patient, were: levothyroxine 200 µg every morning for previously diagnosed hypothyroidism; selegiline 20 mg every morning as an off-label adjunctive antidepressant strategy for treatment-resistant depression; vilazodone 20 mg every morning as the primary antidepressant; gabapentin 1200 mg at bedtime for sleep continuity and comorbid anxiety; and midazolam 15 mg at bedtime as a sedative-hypnotic for severe insomnia. All non-DORA psychotropic doses had been stable for at least four weeks before lemborexant initiation. She denied alcohol and illicit drug use. There was no exposure to strong or moderate CYP3A modulators listed as clinically meaningful in the lemborexant label. 2

On day 5, she awoke with sudden, intrusive suicidal thoughts that she described as qualitatively new and more severe than prior ideation. She denied having a specific plan but reported overwhelming intent that frightened her and led her to present to the emergency department. Vital signs and physical examination were unremarkable. Mental state examination showed anxiety, intact reality testing, and suicidal ideation with intent but no plan. There were no signs of akathisia, mania, psychosis, or melancholic depression. Pill counts and pharmacy records confirmed adherence and dosing, and no other medication changes had occurred. Although formal depression severity rating scales (e.g., HAM-D or MADRS) were not administered at the index emergency presentation given the acute clinical context, the patient's pre-event mood state had been longitudinally documented in routine outpatient records and was reported as unchanged from the days preceding lemborexant initiation. The patient consistently described the suicidal ideation as qualitatively distinct from her chronic depressive experience: intrusive, sudden, and dissociated from her baseline mood. No observable worsening of depressive symptoms (psychomotor changes, anhedonia, hopelessness, guilt, or somatic symptoms) was reported by the patient or documented by the treating team in the days surrounding the event.

Complete blood count, comprehensive metabolic panel, thyroid-stimulating hormone, pregnancy test, and urine toxicology were unremarkable. Alternative explanations considered included acute depressive worsening, a mixed or hypomanic state, psychosis, akathisia, substance intoxication, metabolic or endocrine disturbance, and a pharmacokinetic interaction increasing lemborexant exposure; none were supported by the clinical assessment.

Application of the Naranjo Adverse Drug Reaction Probability Scale yielded a total score of 6 ( Table 1 ), corresponding to a probable causal relationship. 9 Lemborexant was discontinued immediately. The patient was admitted to a secure psychiatric unit for 24-hour observation with suicide precautions and safety planning. No additional pharmacological treatment was initiated.

**Table 1:** Naranjo Adverse Drug Reaction Probability Scale Scoring for the Association between Lemborexant and Acute Suicidal Ideation

Question	Answer	Justification	Score
1. Are there previous conclusive reports on this reaction?	No	There are pharmacovigilance signals within the DORA class, but no conclusive reports specifically confirming lemborexant-induced suicidality.	0
2. Did the adverse event appear after the suspected drug was administered?	Yes	Severe suicidal ideation emerged on day 5 after lemborexant initiation.	+ 2
3. Did the adverse event improve when the drug was discontinued?	Yes	Complete remission within 24 hours of discontinuation, without additional pharmacological changes.	+ 1
4. Did the adverse event reappear when the drug was readministered?	Do not know	No rechallenge was attempted for ethical reasons.	0
5. Are there alternative causes that could have caused the reaction?	No	No acute alternative cause was identified after clinical, laboratory, toxicological, and medication review; however, baseline psychiatric vulnerability remains an important background risk factor.	+ 2
6. Did the reaction reappear when a placebo was given?	Do not know	Placebo challenge not performed.	0
7. Was the drug detected in toxic concentrations?	Do not know	No plasma level data available.	0
8. Was the reaction dose-related?	Do not know	Fixed dose (10 mg nightly); no dose variation occurred.	0
9. Did the patient have a similar reaction to similar drugs?	No	No history of prior DORA exposure or similar reactions.	0
10. Was the adverse event confirmed by objective evidence?	Yes	Documented clinical observation confirming suicidal ideation with intent, requiring psychiatric admission.	+ 1
**Total**		Probable adverse drug reaction (≥ 5)	**6**

Scoring: ≥ 9 = definite; 5–8 = probable; 1–4 = possible; ≤ 0 = doubtful.

Suicidal ideation resolved completely within 24 hours. She subsequently engaged in cognitive behavioral therapy for insomnia. During the following 3 months, suicidal ideation did not recur. Later, ziprasidone was introduced and titrated to 80 mg/day, after which the patient reported sustained improvement in sleep continuity to approximately 8 hours per night. At 6-month follow-up, the patient remained free of suicidal thoughts and maintained functional stability.

## Discussion

The onset within 5 days of the first dose, absence of alternative acute precipitants, and prompt resolution after discontinuation support a probable temporal and clinical association with lemborexant, consistent with a probable adverse drug reaction by Naranjo criteria. The current label advises monitoring for new or worsening depression or suicidal ideation. 2 Pharmacovigilance analyses using the World Health Organization (WHO) spontaneous reporting database have identified suicidality signals for DORAs, and analyses based on the United States Food and Drug Administration Adverse Event Reporting System (FAERS) have produced mixed but clinically relevant findings for individual agents. 10 11 12 This case adds individual-level documentation in a clinically common population, namely, severe insomnia with mood disorder vulnerability.

Notably, recent evidence has suggested that lemborexant can be efficacious and generally well tolerated in patients with mood disorders. The SELENADE 12-week, open-label, multicenter pilot study reported improvements in both insomnia and depressive symptoms in patients with a current depressive episode, with no serious treatment-emergent adverse events; however, given its open-label, pilot design ( *n* = 83), it was not powered to detect rare psychiatric adverse events. 7 Notably, SELENADE explicitly excluded patients with recent manic/hypomanic episodes and with parasomnias or restless legs symptoms, and enrolled a population with relatively mild depressive severity (mean HAM-D-17 11.4–13.9), characteristics that contrast sharply with the present patient (severe treatment-resistant depression with prior parasomnias and a strong family history of mood disorders and suicide). Against this background, several features of the present case are noteworthy: the rapidity of suicidal ideation emergence, its temporal alignment with the expected approach to steady-state lemborexant exposure, and the depth of pre-existing treatment resistance, including prior exposure to ketamine, electroconvulsive therapy, and repetitive transcranial magnetic stimulation. Together, these elements may help generate hypotheses about a potential high-risk profile, that is, patients with severe, multiply refractory mood disorder, profound chronic insomnia, and family history of mood disorders and suicide, in whom DORA-related suicidality signals might warrant particular vigilance, even when group-level data remain reassuring.

The observed time course is compatible with known lemborexant pharmacokinetics. Peak concentrations occur ~ 1 to 3 hours after dosing, with an effective half-life of ~ 17 to 19 hours. 2 3 Lemborexant is metabolized via CYP3A, and its major circulating metabolite (M10) retains orexin receptor affinity. 2 3 13 Steady-state exposure is achieved within several days, consistent with symptom onset on day 5.

At the systems level, several converging mechanisms could plausibly link acute orexin receptor blockade to the rapid emergence of suicidal ideation in a vulnerable patient. Orexin (hypocretin) neurons originate in the lateral hypothalamus and project diffusely to monoaminergic arousal nuclei, including the locus coeruleus (noradrenaline), dorsal raphe (serotonin), and ventral tegmental area (dopamine), as well as to cortical regions involved in affective regulation and behavioral control. 14 15 Through these projections, orexin signaling normally supports arousal, motivational salience, stress reactivity, and reward processing. 16 Acute dual blockade of OX1R and OX2R may therefore destabilize these networks through three non-mutually exclusive pathways: (i) abrupt suppression of orexin tone within stress-related circuits (paraventricular nucleus, amygdala, locus coeruleus), with paradoxical effects in individuals with hyperreactive stress systems 16 ; (ii) sudden modulation of monoaminergic tone, particularly serotonergic and dopaminergic, with secondary effects on prefrontal–striatal circuits implicated in impulse control and value-based decision-making 17 18 ; and (iii) acute changes in sleep–wake regulation and REM-related affective processing, which may amplify negative affect and intrusive cognition; we note that post-treatment polysomnography was not performed and these mechanisms remain hypothetical. In a patient with severe pre-existing insomnia (sleep efficiency 14.5% with absent REM sleep on pretreatment polysomnography) and high familial loading for mood disorders and suicide, such destabilization could plausibly contribute to the emergence of intrusive suicidal ideation independent of clinically evident mood change. The patient's emphasis on the intrusive and qualitatively novel character of the ideation, without observable mood worsening, is consistent with this mechanistic framework, although confirmation will require dedicated mechanistic and pharmacovigilance studies.

Whether the reaction observed here is specific to lemborexant or reflects a class effect of DORAs remains uncertain. Suicidality signals have been described for the DORA class as a whole in WHO and FAERS analyses, although effect estimates differ across individual agents and are subject to important reporting biases. 10 11 12 In the present case, rechallenge with lemborexant or a switch to another DORA (e.g., suvorexant or daridorexant) was not attempted, given the severity of the reaction, the availability of non-DORA strategies (cognitive behavioral therapy for insomnia and, subsequently, low-dose ziprasidone), and the ethical implications of re-exposure. Until comparative head-to-head safety data become available, the decision to switch to another DORA after a probable DORA-related psychiatric adverse event should be made cautiously and on a case-by-case basis, ideally as part of shared decision-making with the patient. Looking forward, selective orexin-2 receptor (OX2R) antagonists currently in clinical development, such as seltorexant, may offer a different pharmacological profile. In a recent phase 2 randomized study, adjunctive seltorexant in patients with major depressive disorder showed favorable tolerability and preliminary antidepressant efficacy versus quetiapine extended-release, particularly at the 20 mg dose and among patients with prominent baseline insomnia symptoms. 19 Whether selective OX2R antagonism carries a different psychiatric safety profile than dual orexin blockade is an important open question that will require dedicated long-term safety and post-marketing pharmacovigilance evaluation.

No concomitant medications known to be strong or moderate CYP3A inhibitors were identified, making a pharmacokinetic interaction increasing lemborexant exposure unlikely. A pharmacodynamic interaction between lemborexant and the patient's concomitant benzodiazepine (midazolam) is, however, a more plausible contributing mechanism. Both agents depress arousal-related networks, lemborexant through dual orexin receptor blockade and midazolam through GABA-A receptor potentiation, and their combined action on cortical arousal, REM-related affective processing, and impulse-control circuits could plausibly amplify the destabilizing effects discussed above. No change in benzodiazepine dose occurred around the event; the chronic baseline benzodiazepine exposure could nonetheless have predisposed the patient to a more abrupt response to DORA initiation. The critical observation is that suicidal ideation remitted fully after stopping the DORA while the benzodiazepine was continued, supporting lemborexant as the most likely proximate factor in this patient.

This report has limitations. Rechallenge was not attempted for ethical reasons, serum drug concentrations were unavailable, and there were no dose–response data. In addition, formal depression severity rating scales (e.g., HAM-D or MADRS) were not administered at the time of acute presentation; clinical assessment relied on structured psychiatric evaluation and longitudinal outpatient documentation rather than standardized scoring. As a single case, generalizability is also limited, and the findings should be regarded as hypothesis-generating and interpreted alongside the emerging class-level pharmacovigilance signals for DORAs. 10 11 12 Although no dose changes occurred around the event, concomitant central nervous system depressants (midazolam and gabapentin) may have influenced sleep–wake physiology or clinical presentation, and a contribution to the event cannot be fully excluded. These findings suggest practical precautions when initiating a DORA: document a structured suicide risk assessment, counsel patients and families to report new or worsening suicidal thoughts promptly, arrange early follow-up within 1 to 2 weeks, and discontinue the drug immediately if suicidal ideation emerges.

## Conclusion

This case suggests that lemborexant may contribute to the emergence of acute suicidal ideation in susceptible individuals. Careful early monitoring, structured suicide risk assessment, and prompt withdrawal if suicidal ideation emerges are prudent measures, particularly in patients with mood disorders.
